# Minor heavy metal: A review on occupational and environmental intoxication

**DOI:** 10.4103/0019-5278.44692

**Published:** 2008-12

**Authors:** Viroj Wiwanitkit

**Affiliations:** Wiwanitkit House, Bangkhae, Bangkok, Thailand 10160

**Keywords:** Heavy metal, intoxication

## Abstract

Heavy metal is widely used in industries and presents as a problematic environmental pollution. Some heavy metals, especially lead and mercury, are well described for their occupational and environmental intoxication whereas the other minor heavy metals are less concerned. In this article, the author will present the details of occupational and environmental minor heavy metal intoxication. This review focuses mainly on aluminum, tin, copper, manganese, chromium, cadmium and nickel.

## INTRODUCTION

Heavy metal is widely used in industries and presents as an important environmental pollution. Heavy metal intoxication is a new public health threat in the present day. Some heavy metals, especially lead and mercury, are well described for their occupational and environmental intoxication whereas the other minor heavy metals are less concerned. In this article, the author will review the details of occupational and environmental minor heavy metal intoxication. This review focuses mainly on aluminum, tin, copper, manganese, chromium, cadmium and nickel.

## ALUMINUM INTOXICATION

### Occupational aluminum intoxication

There are many reports of respiratory disease according to aluminum exposure. Occupational aluminum intoxication is important in occupational medicine. For factory workers in the UK and Germany, pulmonary disease due to respirable aluminum particulates is compensated as a workplace disability.[1] Repeated periodic fever of the aluminum factory worker due to intoxication is also mentioned.[2] Of interest, it has been implicated that aluminum is involved in the etiology of Alzheimer's disease and other neurodegenerative disorders, although this is controversial.[3] Sińczuk-Walczak *et al*.[3] performed a study to assess the effects of Al on the nervous system's functions in workers chronically exposed to this metal. According to this work, Sińczuk-Walczak *et al*.[3] suggested that exposure to aluminum oxide at concentrations within the Maximum Admissible Concentration (MAC) values induces subclinical effects on the nervous system. White *et al*.[4] also supported the existence of a syndrome characterized by incoordination, poor memory, impairment in abstract reasoning and depression in a similar observation in 25 workers from an aluminum smelting plant. Therefore, there is no doubt for the necessity of biomonitoring for the risk faced by workers. Urinary fluoride is presently accepted as an exposure index for aluminum.[5]

There are also some interesting reports on cutaneous abnormalities in workers exposed to aluminum. Skin telangiectasia in workers of an aluminum processing plant was studied by Balić and Kansky in 1988.[6] Balić and Kansky[6] assumed that telangiectases were caused by hydrogen fluoride and other fluorides. Working in the current environment and wearing masks might protect young subjects from developing the lesions.[7] Prevention of bony fluorosis in aluminum smelter workers becomes the present focus.[8]

### Environmental aluminum intoxication

Environmental aluminum contamination is widely mentioned. Zaida *et al*.[9] studied the level of lead and aluminum in infants’ hair, diet and the local environment in the Moroccan city of Marrakech and found that the mean values in the childrens’ hair were 6.6 and 9.5 μg/g for lead and aluminum, respectively. According to this work, age, gender and the parents’ occupations did not impact on aluminum contents.[9] Zaida *et al*.[9] proposed that the higher value for aluminum compared with lead can be explained by the higher levels of aluminum available in both the infant food and the environment. Souad *et al*.[10] performed another study to determine the level of aluminum contamination in infant hair and diet and in the local environment of the Moroccan city of Marrakech. Souad *et al*.[10] concluded that during weaning, beverages like tea, widely used in Morocco, represent an important source of aluminum contamination and that the aluminum content in drinking water was also above the international standard.

At present, environmental surveillance of aluminum in surface water is performed in many countries. Guibaud and Gauthier[11] performed a study of aluminum concentration and speciation of surface water in four catchments in the Limousin region, France. According to this work, despite high concentrations of total aluminum at low pH, the monomeric toxic forms of aluminum, computed with a speciation software, were always inferior to the toxic values for fish.[11] Guibaud and Gauthier[12] also reported that if the sum of the concentrations of Al^3^+, Al(OH)^2^+ and Al(OH)^4^- was taken into consideration, the concentration of aluminum recorded might have adverse effects on aquatic life in the upstream catchment of the river Vienne.

## TIN INTOXICATION

Tin miners are concerned with particular attention to lung cancer and exposure to radon, not the tin.[13] However, Chen and Chen,[14] who studied tin mines in China, reported that exposures to radon were low in the four tin mines and that no carcinogenic polycyclic aromatic hydrocarbons could be detected.

## COPPER INTOXICATION

### Occupational copper intoxication

Occupational copper intoxication is a great concern in occupational medicine.

For example, an instrumental neutron activation analysis was performed to assess the exposure degree of a worker group from a copper smelter by Tshiashala *et al*.[15] According to this work,[15] an excessive high concentration of copper was observed in the investigated group. Sulotto *et al*.[16] studied copper exposure in a group of 68 industrial welders. According to this work, serum copper was higher in workers exposed full time than in those exposed part time.[16] Finally, the exposure characterization focusing on the concentrations of copper in the inhalable aerosol fractions as well as in the water-soluble and water-insoluble subfractions in a copper refinery factory was assessed by Thomassen *et al*.[17] According to this work, for the pyrometallurgical operations, a comparison indicated that water-soluble copper levels were on average 19-fold higher compared with nickel.[17] Nieboer *et al*.[18] determined the urinary copper concentrations for 127 copper refinery workers. No gender difference was observed for copper.[18] Based on the inhalable aerosol levels reported previously for the same subjects, the observed urinary copper concentrations were significantly lower than expected.[18]

### Environmental copper intoxication

Environmental copper intoxication, as reported for humans, is limited. However, a well-known environmental copper intoxication in animals was reported in 1991 by Gummow *et al*.[19] The outbreak occurred among cattle on a farm in north-eastern Transvaal.[19] In this report, the pathological findings and the liver and kidney analyses confirmed that the cattle had died of chronic copper poisoning.[19] The problems of the wild animals of Africa are also documented.[20]

## MANGANESE INTOXICATION

### Occupational manganese intoxication

As mentioned previously, neurological disorder is the focused concern for occupational manganese intoxication. Mental and neurological disorders in chronic poisoning are the most acute symptoms of occupational neurointoxication.[3] The results of neuropsychological studies carried out in different countries and in different occupational groups exposed to low manganese concentrations show the wide range of changes in the function of the nervous system.[3] Chia *et al*.[21] reported an interesting study on the neurobehavioral functions among workers exposed to manganese ore. According to this work, the exposed workers had significantly poorer motor speed, visual scanning, visuomotor coordination, visuomotor and response speed and visuomotor coordination and steadiness, and a clinical examination did not demonstrate any disorder among the two groups.[21] Chia *et al*.[21] concluded that a neurobehavioral test battery might be a more sensitive method than a clinical examination in detecting early changes in the motor function among manganese-exposed workers. Zheng *et al*.[22] demonstrated that the cumulative exposure of manganese of about 1 mg/m3 per year might induce subclinical signs of intoxication. Finally, Shin *et al*.[23] recently proposed the high signal intensity on magnetic resonance imaging as a predictor of neurobehavioral performance of workers exposed to manganese.

There are also some reported studies on the biomarker for occupational manganese exposure. An assessment based on the combined measurements of blood manganese and air manganese is valid for interpreting the workers’ hazard.[24] Lander *et al*.[24] indicated that blood manganese might be a valuable parameter for estimating recent exposure; however, more information is still needed about the blood manganese level and its relation to neurological symptoms and the concerns of intoxication. Bader *et al*.[25] reported the biomonitoring of manganese in blood, urine and axillary hair following low-dose exposure during the manufacture of dry cell batteries. Bader *et al*. concluded that blood manganese was a specific and suitable parameter for the biomonitoring of MnO2 exposure, although its validity is limited to group-based calculations, whereas urinary manganese failed to allow a differentiation between exposed workers and referents. Bader *et al*.[25] also proposed that the suitability of manganese analysis in hair for biomonitoring purposes suffered from a relatively great background variation as well as from analytical problems.

### Environmental manganese intoxication

The effects of manganese in air are widely concerned.[26] Adkins *et al*.[27] studied an acute inhalation exposure of laboratory mice to respirable Mn3O4 aerosols and found that a systemic distribution of the manganese was observed in various tissues following exposure. Another present concern is the use of the gasoline additive methylcyclopentadienyl manganese tricarbonyl, which is documented for the generation of air manganese and can be the future problem.[26]

## CHROMIUM INTOXICATION

### Occupational chromium intoxication

There are some reports on the occupational chromium intoxication in workers at risk. Conroy *et al*.[28] studied the cadmium exposure during abrasive blasting. Conroy *et al*.[28] reported that airborne levels in the containment exceeded the Occupational Safety and Health Administration's permissible exposure limits by factors of 3.1. Boscolo *et al*.[30] also studied the effects of chromium on the lymphocyte subsets and immunoglobulins from the normal population and exposed workers. Boscolo *et al*.[30] suggested that trivalent chromium might be involved in mechanisms regulating the immune response in humans. Exposure to chromium has a significant effect on the immune system. Therefore, it is evident that worker exposure to chromic acid in the electroplating workplace should be reduced to a minimum.[31]

As mentioned previously, chromium is also strongly related to cancer. The content of chromium was determined in the organs of six chromate workers who had worked in a chromate chemical manufacturing plant, had been exposed to a high amount of chromium for over 10 years and had died of lung carcinoma.[32] Kishi *et al*.[32] reported that it was apparent that the metal remained in the lungs long after exposure to chromate had ceased. The concentration of chromium in the upper lobes is usually significantly higher than that in the lower lobes, suggesting regional differences either in clearance from or deposition in the lung.[32]

### Environmental chromium intoxication

There are some reports on environmental chromium intoxication. The present focus is on chromium as a contaminant in the big cities. In 2005, Chillrud *et al*.[33] reported steel dust in the New York city subway system as a source of chromium exposure for transit workers. Elevated airborne exposures of teenagers to chromium could be detected.[33]

## CADMIUM INTOXICATION

### Occupational cadmium intoxication

There are many reports on nickel intoxication in industrial workers. In 1982, Chan *et al*.[34] studied workers exposed to cadmium in an alkaline storage battery manufacturing and polyvinyl chloride (PVC) compounding plant. According to this work, urine cadmium excretion in workers exposed to cadmium in alkaline storage battery manufacturing and PVC compounding increased with working time and there was also a good correlation between the blood and urine cadmium levels.[34] Similar results were also reported by Dewell[35] and Hassler *et al*.[36] in alkaline battery workers. Indeed, a statistically significant relationship between olfactory impairment and cadmium concentration in blood, urine and the workplace air was observed by Rydzewski *et al*.[37] However, such a relationship was not determined with regard to the duration of work.[37] Rydzewski *et al*.[37] indicated the need to carry out routine olfactometries. Marek *et al*.[38] concluded that cadmiumuria indicated a greater dependence on the degree of exposure than did the concentration of this metal in blood. The findings suggest that the cadmium concentration in the blood and urine after the body stores it to saturation is a better informant of current exposure than of cadmium stored in the organism. According to the dose–response relationship, an increase of low molecular protein excretion in urine can be seen in one-tenth of the cases at cadmium in urine, amounting to 10–15 μg/g creatinine and cadmium in blood years of about 300–400.[39]

Follow-up of biological monitoring results in cadmium workers removed from exposure was assessed by McDiarmid *et al*.[40] In this work, while the biological monitoring parameters for most workers significantly declined during the 18 months of medical removal, the biological parameters for only a worker's values returned to the normal range.[40] In another study by Chan *et al*.,[41] the lung function status of workers in a cadmium–nickel battery factory was reexamined 3 years after the first study, which showed a mild restrictive effect. According to this work, the blood and urine cadmium concentrations were significantly lower than previously, consistent with the decreased cadmium-in-air levels, and the rate of respiratory symptoms decreased particularly in the workers who were no longer exposed to cadmium.[41] McDiarmid *et al*.[40] noted that significant policy implications of medical removal protection beyond the current 18-month period provided by the cadmium standards exist and require physician discretion.

### Environmental cadmium intoxication

The degree of cadmium contamination in wildlife can be used as an indicator for the environmental monitoring of cadmium poisoning.[42] For example, wild pigeons were mentioned as a specific medium of monitoring for evaluation of cadmium pollution in some urban areas.[43] The best situation of environmental cadmium intoxication in the global record is in Japan, namely Itai-Itai. Cases were first recorded from as early as 1929, increased rapidly to the peak in 1955–1959 and rapidly decreased up to the 1970s, and it was found that the later the patient was born, the younger was the age of onset, although there was no difference of ages of onset between the cases born in the 1910s and those born in 1920.[42] Cadmium concentrations in blood and urine are significantly higher in the Itai-Itai disease patients, suspected patients and inhabitants of cadmium-polluted areas.[44] Uetani *et al*.[45] studied tissue cadmium concentrations of people living in a cadmium-polluted area in Japan. Uetani *et al*.[45] demonstrated that the tissue cadmium concentrations of some inhabitants in cadmium-polluted areas other than the Jinzu river basin were equal to those of the patients with Itai-Itai disease, and that patients with Itai-Itai disease were present even in these areas. Of interest, in spite of the fact that environmental monitoring did not reveal a significant contamination of the selected areas by cadmium, the urine cadmium levels confirmed that the population living in these areas is really exposed to cadmium.[46] A significant association was detected between urinary beta 2-microglobulin and mortality in a 9-year follow-up study of 3178 cadmium-exposed inhabitants using Cox's proportional hazards model.[47] Liu[48] recently studied cadmium concentrations in hair, urine and blood among residents in a cadmium-polluted area on a 18-year follow-up after soil replacement. According to this work, it was suggested that the body burden influenced the levels of blood cadmium as well as urine cadmium many years after cadmium exposure had decreased.[48]

## NICKEL INTOXICATION

### Occupational nickel intoxication

There are many reports on nickel intoxication in industrial workers. A high risk of respiratory cancer is mentioned for some specific groups of nickel-exposed workers.[49] It is clear, however, that not all forms of nickel exposure are implicated in these excess risks.[49] Urine nickel is the most widely used biomarker for occupational nickel exposure at present. A strong correlation between the level in atmosphere and the urine nickel level is mentioned.[50] Hassler *et al*.[51] studied the urinary and fecal elimination of nickel in relation to the airborne nickel in a battery factory. According to this work, a significant correlation was found between nickel in air and fecal nickel, and smoking habits did not seem to influence either urinary or fecal nickel concentrations.[51] Kiilunen *et al*.[52] performed an occupational hygiene survey in 38 nickel plating shops in Finland. According to this work, the correlation between the concentrations of nickel in the air and in the urine was low and the amount of nickel excreted in the urine exceeded the calculated inhaled amounts.[53] However, Kiilunen *et al*.[53] reported that the frequency of micronucleated epithelial cells in the buccal mucosa of the nickel refinery workers was not significantly elevated by comparison with referents. Kiilunen *et al*.[53] also noted that no relationship was observed between micronucleus frequencies and levels of nickel in air, urine or blood. Of interest, fluctuations of nickel concentrations in urine of electroplating risk workers were also reported.[54] Bernacki *et al*.[54] recommended that nickel analyses of 8-h urine specimens should be used routinely to monitor occupational exposures to nickel, and analyses of end-shift urine specimens are the best alternative in situations where timed urine collections are impractical. At present, the occupational exposure limit for nickel in urine is accepted at the level of 30 μg/L.[55]

### Environmental nickel intoxication

Environmental nickel intoxication is mainly concerned with drinking water. Nickel is a common cocontaminate with lead and copper.[56] The chronic nickel intoxication in doses of 0.5 mg/kg was accepted to lead to disorders in protein metabolism and reduction of body weight, in doses of 0.005, 0.05 and 0.5 mg/kg to disorders in carbohydrate metabolism, hyperhemoglobinemia and erythrocytosis.[57] The atherogenic effect of nickel entering the body in drinking water has also been mentioned for a long time.[58]
